# Clinical exercise physiology students learning with older adults: an innovative simulation-based education programme

**DOI:** 10.1186/s41077-016-0012-3

**Published:** 2016-04-02

**Authors:** Louise Horstmanshof, Robert G. Lingard, Sonja Coetzee, Louise P. Waddell

**Affiliations:** School of Health and Human Sciences, Southern Cross University, PO Box 157, Lismore, 2480 NSW Australia

**Keywords:** Primary Healthcare, Project Leader, Clinical Placement, Clinical Supervisor, Residential Care Facility

## Abstract

In this paper, we report on a series of placements for clinical exercise physiology students in a simulation-based education environment with older, independent adults. The purpose of these placement opportunities was to help prepare students to work confidently and competently with older adults in primary healthcare settings. The effectiveness of these placements was measured through semi-structured interviews with the students, their supervisors and the volunteer patients, and also by analysing the content of the students’ written reflection assignments. A combination of directed content analysis, informed by the research objectives and imposed upon the data, and conventional content analysis, in which codes were developed from themes emerging from the data, was adopted. Coding was based on units of meaning. Overall, the placement aims were met. Students reported increased confidence in communicating with older adults and in using the tools of their trade. This innovative simulation-based education experience helped students gain an understanding of their developing professional identities. However, the data show that some students still failed to recognise the value and importance of communication when working with older adults. The older adults reported that they enjoyed interacting with the students and believed that they had helped the students gain a positive impression of the cognitive and physical abilities of older adults. These older adults had also gained insight into the benefits of exercise physiology in terms of their own wellbeing.

This paper demonstrates the benefits of engaging community support in developing healthcare workers and provides guidelines for replication of these innovative simulation-based education experiences. The paper is limited to reporting the social and community engagement benefits for older adults and the learning opportunities for the clinical exercise physiology students. Further research is needed to demonstrate the health gains for older adults who participate in such programmes.

Clinical exercise physiology (CEP) is an emerging healthcare profession that has seen significant growth over the past decade [1]. University-trained CEPs are recognised nationally by Exercise and Sports Science Australia (ESSA) as accredited exercise physiologists. A CEP graduand is eligible to apply to ESSA for such recognition. CEPs utilise exercise to prevent and manage injury, disability and chronic disease, thus performing a vital role in primary healthcare. Participation in clinical placements and gaining competence while accruing clinical practicum hours is an accreditation requirement for CEPs in Australia. CEP students are required to complete a minimum of 500 h of practicum of which 360 h must be with patients with chronic or complex health conditions [2]. This innovative simulation-based education (SBE) project was designed to address some of the many challenges associated with meeting these clinical placement requirements. Engagement with a community of independently living older adults provided an innovative and authentic SBE environment for these CEP students close to graduation, with unexpected benefits for both groups.

An important aim of this project was to engage students with older adults in a simulated clinical environment. Older Australians are major consumers of general practitioner (GP), nursing and other allied health services. Reforms by the Department of Health and Ageing [3] are aimed at improving primary and preventative care for older people. The purpose of these measures is to keep older people out of the hospital system by enabling older people to maintain their own health and remain within their communities. These reforms recognise older Australians’ preferences to live in their homes for as long as possible and reflect a shift to person-directed care. This is particularly relevant for older persons living independently (classified as independent living), granting them greater autonomy regarding their healthcare choices.

An ageing population and increasing need for quality primary healthcare for older adults necessitates CEP students receiving relevant, practical training and opportunities to develop positive attitudes towards working with older adults. In particular, it is important for students to gain greater understanding of older adults, not living in institutionalised settings, who independently manage their own healthcare.

A primary objective of this project was to provide CEP students with opportunities to interact with older adults currently managing their healthcare independently. Learning experiences where students engage with a diverse range of older adults promote awareness of ageist stereotypes and the negative impacts these stereotypes have on quality healthcare delivery. This innovative learning experience was anticipated to develop students’ communication and interpersonal skills and provide them with an opportunity to work with independent older adults to broaden their understanding and experience of adults aged 65 years and older.

A consequence of ageing is frailty and reduced physical functioning, which can lead to a loss of independence and increased risk of chronic diseases. Research indicates, however, that the association between health status and age is more variable than is often assumed, as many chronic conditions are preventable (or can at least be postponed) and are not an inevitable consequence of ageing [4]. Strategies for delaying the onset of chronic debilitating disease include the prevention of risk factors and the reduction of the prevalence of risk factors (e.g. smoking, lack of exercise, weight gain) before disease develops. After onset of chronic disease, the strategies should shift towards the prevention of progression of disease (e.g. preventing second heart attacks or the complications of diabetes) and reduction of morbidity from disease, or any complications that have already developed [5].

Physical activity is widely acknowledged as a key intervention strategy to preserve and improve physical and mental health in older adults [6–8], with the American College of Sports Medicine recommending aerobic, muscle strengthening and flexibility exercises for older people [9, 10]. Moreover, additional exercises, specifically to improve balance, agility and proprioception, are recommended to reduce the risk of falls and for those with mobility problems [10–12]. Overall, exercise slows down the physiologic changes associated with ageing, promoting cognitive health and complementing chronic disease management [9, 13]. It has also been shown to reduce the rate of falls and to slow deterioration in the ability to perform activities of daily living [14–17]. The ability to self-manage activities of daily living is positively associated with the quality of life [18, 19].

The evidence for the multifaceted benefits of physical activity is compelling. Nevertheless, the physical activity behaviour of most elderly people does not comply with current guidelines [20–22]. Several international studies investigated barriers to physical activity for the elderly, with injury or disability, fear of injury, poor health, lack of time and environmental considerations such as convenience or access, cost and lack of knowledge and motivation being some of the commonly mentioned impediments to increased physical activity [23–28]. These barriers can be addressed with the help of qualified professionals, like CEPs [9, 29].

## Background/context

Increased professional membership and accreditation requirements heighten the demand for clinical placements for CEP students [1]. Meeting this demand and satisfying ESSA’s accreditation requirements is a significant challenge for universities [2]. The use of simulated clinical practice outside of the university environment is one solution proposed to address this growing need [30]. There is growing recognition that SBE can provide a “realistic and flexible alternative to traditional clinical training” ([31] p.1). Better student preparation can be achieved through practical experiences [32], including simulated experiences and assessment of students’ skills and knowledge [33]. SBE has been used effectively in training for a number of allied health professions [34–38].

**Fig. 1:**
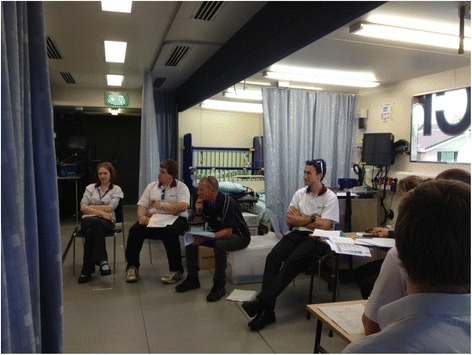
CEP students gather for a debriefing session with their clinical supervisor after the day’s appointments with expert patients (image shared with approval from participants)

SBE can complement the clinical training of CEP students by providing a safe environment for students to develop their skills without exposing clients to unnecessary risk. SBE may be particularly beneficial for CEP students because, in contrast to other allied health professionals using passive therapies (e.g. speech therapy) or a combination of passive and active therapies (e.g. occupational therapy), CEPs consistently use active therapies [2]. These active therapies may be associated with an element of risk for clients: SBE, therefore, offers a particularly safe way for CEP students to develop clinical skills [33]. SBE modalities appropriate for CEP training include case studies, computer simulations, part task training, role plays and, as reported here, an innovative SBE experience of active engagement with expert patients (Fig. 1).

The term “expert patient” has been used in the literature in a variety of ways, for example, for patients who are well-informed about their health conditions [39] or for patients who expertly manage their own chronic health conditions and inform or assist others suffering similar conditions [40]. In the current innovative project, the term was used to describe older adults who independently managed their own healthcare, in contrast to older people in high care settings. It was recognised that these expert patients had great experience and wisdom with respect to their own health experiences and that CEP students could benefit from the sharing of this valuable information.

Typically, allied health students lack knowledge about ageing and have negative attitudes towards working with older adults [41, 42] because many students’ experiences of working with older adults are limited to hospitals and high care settings. Hobbs et al. [42] suggest that novice practitioners’ expectations of particularly healthy older people may be too low. These expectations might lead to the provision of inadequate advice and treatment goals for these clients. Flood and Clark [41] suggest that all nursing programmes provide opportunities to work with older adults in order to help reduce “generational stereotypes and boundaries and ensure adequate preparation for future caregivers of this population” (p. 594). Physiotherapy students at the University of Sydney undertake an aged care component as part of their community health clinical placement to improve their knowledge of and attitudes towards working with older adults [42]. Similarly, CEP students were expected to benefit from learning opportunities involving older adults and were therefore given opportunities to interact with a diverse group of expert patients from independent living facilities, who volunteered to participate. Interaction with relatively healthy older adults was expected to give CEP students a broader perspective of ageing and healthcare. Engaging with older adults in natural, clinical and home settings, rather than in institutionalised environments, is consistent with the primary healthcare emphasis on health promotion and prevention.

Learning is most effective when it occurs in real clinical contexts, including simulated settings, allowing students to apply their theoretical knowledge and skills to actual clinical situations [43]. Such learning environments help bridge the gap between theory and practice, providing students with meaningful and relevant experiences that inform future practice. According to experiential learning theory, learning is most effective when based on experience [44]. This is a cyclical process wherein students engage in new experiences, reflect on these experiences and relate them to their current theoretical knowledge to their plan for the application of new knowledge and skills in further experiences. Rolfe and Sanson‐Fisher [45] suggest that students be exposed to clinical conditions and activities similar to those they are likely to encounter as practising clinicians. Additionally, an environment that fosters a feeling of security for students is essential for effective teaching and learning [46].

The learning outcomes for these placements were that students should be able to:

- Integrate theory with practice in service delivery environments;
- Interact professionally and ethically with exercise physiology clientele and a range of professional colleagues;
- Critically reflect on alignment of own practice with common modes of exercise physiology practice;
- Critically reflect on own practice including identification of strengths and weaknesses.

The aim of this paper is to report how this innovative SBE contributed to students’ clinical placement learning outcomes.

## Method

### Preparation for the placements

Prior to the placements, students and clinical supervisors participated in three workshops, led by the project leader (LH) that addressed communicating effectively with older people, primary healthcare and interprofessional teamwork. Opportunities for interprofessional teamwork were provided through interactions with the staff working in the facilities as well as the nursing and occupational therapy students who also participated in this SBE activity (reported separately). Students were welcomed to the residential facilities and independent living village and participated in orientation tours. The project leader explained the placement project and invited all participants to contribute to the evaluation of this innovative SBE through interviews and, in the case of the students, the mandatory reflection assessment task associated with their clinical placements. In this assessment task, students provided written reflection of 200 words on their progress against the learning outcomes for each placement opportunity.

Participation in the placements was voluntary and involved some choice for the students. The first two placements were of two weeks’ duration, approximately 2 months apart. The third placement was of 1 week’s duration and took place at a sister facility in another town. The final placement took place over a month at the first establishment and gave the students who had participated in the first two placements an opportunity to return for 1 day a week to reconnect and review the progress of their clients. The students participated in one (*n* = 3), two (*n* = 1), three (*n* = 4) or all four placements (*n* = 1).

### Role of the expert patients

**Fig. 2:**
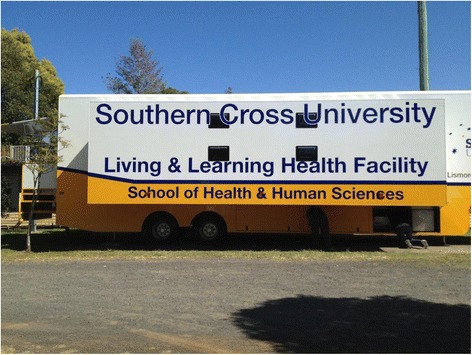
The SCU mobile clinic that was stationed on the premises of the aged care precincts to provide the clinic space for SBE sessions

The expert patients were asked to help prepare allied health students to work in the aged care sector. Flyers that detailed the proposed activities with the students were distributed to the older adults by the participating aged care facilities. The older adults participating as expert patients were also provided with an information session prior to the commencement of this innovative project. The older adults agreed to share their knowledge and experience by attending the University’s mobile health clinic for consultation interviews with students. The majority of consultations took place in the University’s mobile health clinic, which was parked in the grounds of the independent living villages (Fig. 2). However, a small number of consultations were provided on request in the homes of residents. They were also invited to provide feedback on their experiences, in follow-up interviews.

During expert patient consultations (60–90 min in duration), students obtained basic demographic information for each participant and recorded relevant information related to sociocultural issues, mobility problems/ difficulties, the use of mobility aids and personal hygiene issues. They conducted risk and resilience assessments and the Berg Balance assessment [47] and noted any enablers and barriers likely to influence the outcome of the risk assessments, based on primary healthcare principles.

### Data gathering processes

Three different cohorts of participants provided input about the student learning in this project: CEP students from a Master’s programme, expert patients and clinical supervisors. Ten students (seven males and three females) were given placements with two affiliated, independent living aged care facilities in Northern NSW (Australia). At the conclusion of the placement, each student was interviewed by the project leader (LH) and contributed a written reflection that was part of their clinical course assessment. One student was not interviewed and another did not contribute a reflection, resulting in nine interviews and nine reflections for analysis. The 23 expert patients (18 females and five males) also provided their feedback through interviews. Four couples participated in joint interviews, and 15 expert patients were interviewed as individuals, resulting in a total of 19 interviews. Reflections were also contributed by the two clinical supervisors (JM and MR) and by the project leader (LH). Overall, the value of the training in primary healthcare engaging older adults as expert patients and CEP students was assessed, based on 40 documents. Names have been omitted to preserve participant anonymity.

After consultations with the expert patients, students, in groups, discussed assessments, proposed treatments and wrote clinical notes on each case to verify their interpretations and treatments of the expert patients and their conditions. During follow-up interviews with the project leader (LH), students reflected on working with the expert patients and on the training experience in general. The expert patients were approached by the project leader (LH) for interviews. The project leader (LH) also interviewed both clinical supervisors, who had shared supervision of the students across the placements. All interviews were audio-recorded and transcribed verbatim by a member of the project team and were submitted, along with the students’ reflection assessment tasks, for analysis. All interviews were semi-structured for consistency of data collection. Evaluation of this innovation was approved by Southern Cross University’s Human Research Ethics Committee in 2012 (approval number: ECN-12-308).

### Evaluation team

The project leader and interviewer (LH) is a registered psychologist and curriculum development specialist with an interest in healthy ageing. She had not worked with any of the CEP students before. Neither the team member (ML) who transcribed the interviews nor the data analyst (RGL) who undertook the main analysis and interpretation of the evaluation data had ever met any of the participants. They had also not worked in this area of innovation before. One author (SC) worked with these students as their university lecturer and is the only one with in-depth knowledge of clinical exercise physiology. The final author, LW, did not interact with any of the participants.

### Analysis of evaluation data

The transcript documents were subjected to content analysis (CA) after being uploaded as sources into NVivo10 (QSR International), consistent with the methods of Leech and Onwuegbuzie [48]. A combination of directed CA, informed by the learning objectives and imposed upon the data, and conventional CA, in which codes were developed from themes emerging from the data, was applied following Hsieh and Shannon [49]. Coding was based on units of meaning.

A codebook was compiled using a structured definition for each code (RGL). This hybrid structure was developed from the works of other published coders [50–52] and contained six points each: a title; a description that included a “flag” to sensitise the coder to recognise the code in the text; a description of the criteria for including the text; a description of the criteria for excluding the text; an example of the application of the code; and other, relevant notes, such as where the code appeared within a coding hierarchy. An example of a codebook entry is provided in Table 1 to illustrate application of this coding method. Table 2 provides a summary of the coding framework that was established for the CEP student interviews.

**Table 1:** Example of a codebook entry: educating others

Title:	Educating others
Description:	This code is used for statements that related the students’ experiences of teaching others about their own discipline.
Criteria for inclusion:	This code includes only those statements that involved discussion with someone who is NOT working within the CEP discipline.
Criteria for exclusion:	Do not include statements that relate to CEP students educating others in the CEP discipline. Do not include statements that relate to CEP students being educated by others.
Example:	“She [oncolocy nurse] didn’t really know what we did as EPs, and I was explaining it to her…” (Student C)
Notes:	Hierarchy: Benefits of the placement: Educating about own discipline Knowledge acquisition Patient awareness Personal motivation or affirmation Professional insights

**Table 2:** Coding framework, showing coding hierarchy

Rating the experience	
Very good	
Good	
Uncommitted	
Poor	
Not rated	
Placement outcomes	
Attitude to future work with older adults	
Open to possibility	
Interested in another field	
Confirmed decision NOT to work with older adults	
Benefits of the placement	
Educating others	
Knowledge acquisition	
Patient awareness	
Personal motivation or affirmation	
Professional insights	
Skills development	
Unique opportunity experienced	

The evaluation was validated at two levels in this project. First, data triangulation was achieved by using student interviews and reflections, as well as expert patient interviews and supervisor reflections. Second, the coding and analysis of the data by the primary coder (RGL) was interrogated by the evaluation team (LH, SC, and LW) in a half day workshop in which each member of the team trained in the application of the coding framework. Samples of documents were independently analysed by two other academics who are not authors, and definitions were discussed and refined by the team (LH, RGL, SC and LW). Discrepancies were discussed between the original coder (RGL) and the two checkers (LH and SC) until consensus was achieved. Validity of the coding framework was therefore confirmed and the reliability of the coding analysis was verified. Direct quotations, taken from the source documents, are incorporated into this report as further evidence of the reliability of this evaluation.

## Findings

Overall, this innovative SBE experience was regarded as a valuable exercise by the students, with all students rating the experience as “good” or “very good” and being able to list benefits, which will be presented below. Expert patients were also able to speak of the positives they experienced, and when asked, “Would you be happy to have treatment from such students in the future?” Expert patient A, for example, responded affirmatively: “Yes, oh yes! I would definitely come again if I was asked.” The supervisors also affirmed the benefits of the experience with one commenting that he would like to see all students participating in such a programme of learning. While the subjective experience of the project participants was positive, the effectiveness of the engagement with older adults is properly evaluated by reference to the four learning outcomes.

### Hands-on versus information gathering

While the innovative SBE experience clearly provided great scope for students to engage with their theoretical concepts and apply them within the SBE placement, students, supervisors and expert patients noted some limitations. For example, student A, while pleased that she could engage with the “hands-on practical stuff” of her discipline, was also disappointed that so much of the experience involved asking questions and interviewing expert patients. She also noted, pragmatically, that she was unable to try all the things that she had learned because of time restraints on the placement. Student comments generally reflected a valuing of the practical aspects of dealing with patients and a disappointment that interviewing and information-seeking activities were such large components of the exercise, despite this being a primary goal of the project that had been reinforced by pre-placement training. The students’ comments stand in contrast to feedback from the older adults, who were pleased by the communicative aspects of the clinical activities. The expert patients highlighted the need for students to be more confident when working hands-on with the patients and emphasised that the students needed to ask more questions of older adults to truly discern their needs: “If they are going to explore the person, they’re going to need to explore a lot more” (expert patient B). A clinical supervisor also noted a lack of confidence on the part of the students in dealing with the older adults. Clearly, a wide range of skills was practised during the SBE experience; however, at times, a tension was evident between the expectations of the students and the expert patients and between the confidence of the students and the expectations of the supervisors.

### Developing a person-centred approach

Based on their own professional experience, supervisors noted that students were increasingly demonstrating sound clinical reasoning in dealing with older adults as the placement progressed. “[The students] also learnt what essential care is needed as physical and physiological function declines; learning to become more open minded when consulting a resilient population” (clinical supervisor A). Students developed their ability to adapt to the needs and conditions of individual clients. For example, one student learned that for some older adults, the term “gentle movement” was more motivating than the term “exercise.” Another student commented about learning to apply the knowledge and experience gained from working with one client to inform the assessment process with other clients. In this case, the student developed a greater appreciation of the need for modified assessments, for example, the Berg Balance Test. Another student gained valuable experience relating to an unmotivated client. Dealing with older adults presenting with Parkinson’s disease and early onset Alzheimer’s disease was a valuable learning experience in managing unfamiliar situations for another student. Each of these situations, under careful supervision, was an opportunity for the students to demonstrate their discernment and clinical reasoning. Students commented favourably on the constant presence of a supervisor and the immediacy of constructive feedback.

### Primary healthcare

Working according to the principles of a Primary Healthcare model (discussed previously) was also an important part of this innovative SBE experience. This included a focus on individuals and accepting that the patients have important knowledge about their own health needs. Another outcome of the SBE experience was that seven of the nine students reported that they benefited in the area of patient awareness and developed a greater understanding of older adults as a diverse group. The clinical supervisors’ reflections confirmed this observation.

To summarise, the learning outcome of integrating theory and practice in a service delivery environment was clearly achieved through this SBE experience. Students were able to identify a wide range of clinical skills that they employed during this placement and demonstrated sound clinical reasoning and the ability to work according to primary healthcare principles, as was confirmed by the clinical supervisors. However, students were more inclined to value the practical and active aspects of their placement, whereas the expert patients placed greater value on the communicative aspects of their involvement with the students.

### Gaining professional insights

The benefit of the placement that was most frequently noted by students (eight out of nine) was the gaining of professional insights. Professional insights were gained in two distinct areas: the role of the CEP (in working with older adults) and the roles of other professional colleagues. Student B thought he understood his role within the context of aged care, thinking “it would be more like within the nursing home,” but learned that dealing with older adults in independent living situations meant taking a “completely different” approach to exercise prescription. Observing the way that a physiotherapist’s aide went about her work gave student C insight into the different ways that exercise opportunities could be given to older adults.

In addition to CEP students gaining an understanding of the professional milieu in which they were training to work, two students noted that they also had the opportunity to help other students, who were on clinical placement at the same time, to develop their understanding of the role of the exercise physiologist. Student C reported the opportunity to explain to an oncology nurse the role of a CEP, with the outcome that a discussion arose about including a CEP in a health team for oncology patients. The students’ observations demonstrated that they were presenting identities as exercise physiology professionals and had a clearer understanding of the professional landscape for which they were being prepared. Clinical supervisor A affirmed that “[t]he students learnt where the role of an [exercise physiologist] fits into the healthcare system.”

### Verbal repertoire

Evidence of student reflection upon personal practice showed that they had made conscious adjustments to the ways in which they approached the application of their skills. For example, student D provided a more personalised approach to an expert patient by realising that cognitive impairment and functional impairment were different qualities. A similar experience was demonstrated in the way that student E learned to adjust his language to accommodate the meanings expert patients placed on particular words. Specifically, he consciously spoke of “movement” rather than “exercise.” Student F commented on how he felt there was an improvement in his “confidence in being able to identify any problems and issues that might occur in [a patient’s] general life as well as changes during the exercise classes.” Student C also spoke of “not judging a book by its cover” when he realised that a patient initially presenting as well and strong might not be so, and vice versa.

In addition to the formalised learning objectives of this project, other outcomes were noted in the interviews with students and supervisors. Students reported that they had grown in their awareness of older adults as a cohort with whom they might have professional interactions during their careers as CEPs (seven out of nine). Students commented on the wide range of functionality of the older adults and on the range and level of activity in which the older adults were engaged. Further, an element of surprise was evident in the students’ responses to working closely with the independent, older adults, as exemplified in the comments from student G:

"I was just surprised with the self-contained housing around here. I was very surprised. I thought that everyone would be under care. [The elderly] are so nice and friendly, it’s great. They’re not forced to be there, so it’s great. It’s been very good".

### Appreciation of diversity in older adults

The development in the positive attitudes of the students was also noted by the supervisors, with clinical supervisor B affirming that he thinks: “the students are more aware of the different levels of functioning and capacities in this age group […]. This innovative SBE experience has widened students’ experiences; especially that age isn’t a limit.”

Students gained an appreciation of just how independent, functional and healthy many of the older adults were. Comments included, “I guess I was very surprised. I thought everyone would be under care” and “some of the clients surprised me a bit. You look at them on paper, and they’re 86 years old and …supposed to have all these things wrong with them, and then they just wander in like they’re 10 years old.”

A further outcome, linked to a growing awareness and appreciation of the cohort of older adults, relates to the attitude to working with older adults in a future professional capacity. Students reported that they found the placements enjoyable. They particularly enjoyed being able to work with a diverse range of older adults as they had had limited experience with this population during clinical practice sessions at the University Health Clinic. One student commented that it was difficult “to get a chance to work with the healthier side of the elderly,” which this innovative SBE experience enabled. Similarly, another commented that most students had “never dealt with the elderly before.” During the interview, students were asked how the placement had influenced their attitudes towards working with older adults after graduation. Of the eight students who responded to this question, six indicated that they were open to the idea of working with older adults: one indicated that he had already decided not to work with older adults and that the placement had not changed his mind. The eighth student stated that though he was “open-minded” he already had an interest in another field. Overall, a positive attitude to the prospect of working with older adults was expressed by these students. This is an important outcome for this project, which aimed to promote an awareness of ageist stereotypes and developing positive attitudes towards working with older adults.

## Discussion

Overall, this innovative SBE experience satisfied the needs and goals of the multiple stakeholders involved in the project. In terms of the project’s objectives, the students were afforded the opportunity to interact with older adults in ways that encouraged integration of theory with practice, enhanced their communication skills and enabled interprofessional teamwork discussions in primary healthcare settings. The management and staff of the independent living villages and associated residential care facilities welcomed the opportunity for their staff, residents and clients to gain an understanding of clinical exercise physiology at work. Additionally, it was a means of providing further education on preventative healthcare for these older adults in terms of their self-management through the use of exercise and/or physical activity.

The clinical supervisors were pleased with the progress they saw in their students and appreciated the opportunities and support provided to maximise the learning experiences. Improvements were observed and noted in the levels of knowledge, skills and attitude towards working with older adults. The students reported that they felt that they had developed confidence in working with older adults and had gained a positive impression of older people’s abilities, both cognitively and physically. Students enjoyed having the opportunity to test their skills and adapt their approaches to this diverse group of older adults. In particular, they enjoyed linking theory to practice, applying clinical reasoning in facilitated debriefing sessions, reflecting on the experiences and having supervisors on site for prompt feedback and guidance. This is consistent with other studies that show these elements to be highly ranked by students in terms of building their clinical reasoning abilities [53]. The clinical supervisors also commented on the support and guidance afforded by having the project leader (LH) on site or available each day. The benefits of the learning from this innovative SBE experience will serve these students well in their future healthcare careers. As Prout et al. [32] conclude, experiential and interprofessional learning in the field provides students with “grounded opportunities to develop into effective and reflective practitioners” (p. 158). This outcome addressed the aims of preparing health workers to meet the demands of a growing, older demographic.

This paper is limited to reporting the social and community engagement benefits for the older adults and the learning opportunities for the CEP students. While there is evidence that this innovative SBE experience with older adults contributed positively to the development of CEP students as future clinicians, research is needed to demonstrate the health gains for older adults who participate in CEP programmes. Although the older adults, themselves, said that they had enjoyed interacting with the students and contributing to their learning experiences and skills development, more can be done to ensure that older adults, living in residential care facilities, are able to access CEPs as part of their overall healthcare choices.

CEP is a young discipline with growing acceptance of its place in primary healthcare as a preventative measure [54]. Further research is needed to build on the data gathered during these innovative activities to demonstrate the effectiveness of CEP for older adults, especially in terms of pain management [55, 56] and falls prevention as an active therapy [12, 17, 57]. This will contribute to the growing body of evidence of the effectiveness of CEP for funding consideration in healthcare packages.

## Consent

Written informed consent was obtained from all participants for the publication of this report and any accompanying images.

## References

[1] Sealey R, Raymond J, Groeller H, Rooney K, Crabb M, Watt K (2013). Current practice of clinical exercise physiology placement supervision in Australia: 2013 report.

[2] Selig S, Torode M, Otago L, Pascoe D, Charity M, Raymond J, et al. Final report: curriculum renewal in exercise science. Strawberry Hills, NSW 2012, Australia: Australian Learning and Teaching Council, 2011

[3] Department of Health and Ageing (2012). Living longer. Living better.

[4] Khaw KT (1997). Epidemiological aspects of ageing. Philos Trans R Soc Lond B Biol Sci.

[5] Fries JF, Bruce B, Chakravarty E (2011). Compression of morbidity 1980–2011: a focused review of paradigms and progress. J Aging Res.

[6] Chodzko-Zajko WJ, Proctor DN, Singh MAF, Minson CT, Nigg CR, Salem GJ (2009). American College of Sports Medicine position stand. Exercise and physical activity for older people. Med Sci Sports Exerc.

[7] Nelson ME, Rejeski WJ, Blair SN, Duncan PW, Judge JO, King AC (2007). Physical activity and public health in older adults: recommendation from the American College of Sports Medicine and the American Heart Association. Circulation.

[8] Physical Activity Guidelines Advisory Committee (2008). Physical Activity Guidelines Advisory Committee Report.

[9] Ehrman JK, de Jong A, Sanderson B (2010). ACSM’s resource manual for guidelines for exercise testing and prescription.

[10] Chou C-H, Hwang C-L, Wu Y-T (2012). Effect of exercise on physical function, daily living activities, and quality of life in the frail older adults: a meta-analysis. Arch Phys Med Rehabil.

[11] Thompson W, Gordon N, Pescatello L (2010). ACSM’s guidelines for exercise testing and prescription.

[12] Gillispie L, Robertson M, Gillispie W, Sherrington C, Gates S, Clemson L (2012). Interventions for preventing falls in older people living in the community. Cochrane Database Syst Rev.

[13] Angevaren M, Aufdemkampe G, Verhaar HJJ, Aleman A, Vanhees L (2008). P2-386: physical activity and enhanced fitness improve cognitive function in older people without known cognitive impairment: a Cochrane systematic review. Alzheimers Dement.

[14] Shimada H, Uchiyama Y, Kakurai S (2003). Specific effects of balance and gait exercises on physical function among the frail elderly. Clin Rehabil.

[15] Halvarsson A, Franzén E, Ståhle A (2015). Balance training with multi-task exercises improves fall-related self-efficacy, gait, balance performance and physical function in older adults with osteoporosis: a randomized controlled trial. Clin Rehabil.

[16] Sherrington C, Tiedemann A, Fairhall N, Close JCT, Lord SR (2011). Exercise to prevent falls in older adults: an updated meta-analysis and best practice recommendations. NSW Public Health Bulletin.

[17] Yamada M, Higuchi T, Nishiguchi S, Yoshimura K, Kajiwara Y, Aoyama T (2013). Multitarget stepping program in combination with a standardized multicomponent exercise program can prevent falls in community-dwelling older adults: a randomized, controlled trial. J Am Geriatr Soc.

[18] Andersson LB, Marcusson J, Wressle E (2014). Health-related quality of life and activities of daily living in 85-year-olds in Sweden. Health Soc Care Community.

[19] Andersen CK, Wittrup-Jensen KU, Lolk A, Andersen K, Kragh-Sørensen P (2004). Ability to perform activities of daily living is the main factor affecting quality of life in patients with dementia. Health Qual Life Outcomes.

[20] Australian Institute of Health and Welfare (2010). Australia’s health 2010.

[21] Craig R, Mindell J, Hirani V (2009). Health survey for England 2008; Physical activity and fitness.

[22] Haley C, Andel R (2010). Correlates of physical activity participation in community-dwelling older adults. J Aging Phys Act.

[23] de Groot GCL, Fagerströöm L (2011). Older adults' motivating factors and barriers to exercise to prevent falls. Scand J Occup Ther.

[24] Moschny A, Platen P, Klaaßen-Mielke R, Trampisch U, Hinrichs T (2011). Physical activity patterns in older men and women in Germany: a cross-sectional study. BMC Public Health.

[25] Booth ML, Bauman A, Owen N (2002). Perceived barriers to physical activity among older Australians. J Aging And Physical Activity.

[26] Macniven R, Pye V, Merom D, Milat A, Monger C, Bauman A (2014). Barriers and enablers to physical activity among older Australians who want to increase their physical activity levels. J Phys Act Health.

[27] Costello E, Kafchinski M, Vrazel J, Sullivan P (2011). Motivators, barriers, and beliefs regarding physical activity in an older adult population. J Geriatr Phys Ther.

[28] Mathews AE, Laditka SB, Laditka JN, Wilcox S, Corwin SJ, Liu R (2010). Older adults' perceived physical activity enablers and barriers: a multicultural perspective. J Aging Phys Act.

[29] Selig S, Torode M (2008). Meeting the challenges of clinical exercise science and practice: a collaborative university-industry approach.

[30] Selig S, Coombes JS, Otago L, Pascoe D, Raymond J, Torode M (2011). The development of an accreditation scheme for accredited exercise physiologists in Australia. Focus on Health Professional Education.

[31] Health Workforce Australia (2014). Simulated learning environments program. An Australian Commonwealth Initiative [Internet].

[32] Prout S, Lin I, Nattabi B, Green C (2014). ‘I could never have learned this in a lecture’: transformative learning in rural health education. Adv Health Sci Educ.

[33] Hayden JK, Smiley RA, Alexander M, Kardong-Edgren S, Jeffries PR (2014). The NCSBN national simulation study: a longitudinal, randomized, controlled study replacing clinical hours with simulation in prelicensure nursing education. J Nursing Regulation.

[34] Baker C, Pulling C, McGraw R, Dagnone JD, Hopkins‐Rosseel D, Medves J (2008). Simulation in interprofessional education for patient-centred collaborative care. J Adv Nurs.

[35] Bland AJ, Topping A, Wood B (2011). A concept analysis of simulation as a learning strategy in the education of undergraduate nursing students. Nurse Educ Today.

[36] Shoemaker M, Beasley J, Cooper M, Perkins R, Smith J, Swank C (2011). A method for providing high-volume interprofessional simulation encounters in physical and occupational therapy education programs. J Allied Health.

[37] Blackstock FC, Watson KM, Morris NR, Jones A, Wright A, McMeeken JM (2013). Simulation can contribute a part of cardiorespiratory physiotherapy clinical education. Simul Healthc.

[38] Watson K, Wright A, Morris N, McMeeken J, Rivett D, Blackstock F (2012). Can simulation replace part of clinical time? Two parallel randomised controlled trials. Med Educ.

[39] Shaw J, Baker M (2004). “Expert patient”—dream or nightmare?. Br Med J.

[40] Fox J (2005). Care of chronic conditions. The role of the expert patient in the management of chronic illness. Br J Nurs.

[41] Flood MT, Clark RB (2009). Exploring knowledge and attitudes toward aging among nursing and nonnursing students. Educ Gerontol.

[42] Hobbs C, Dean CM, Higgs J, Adamson B (2006). Physiotherapy students’ attitudes towards and knowledge of older people. Australian J Physiotherapy.

[43] Bandali KS, Craig R, Ziv A (2012). Innovations in applied health: evaluating a simulation-enhanced, interprofessional curriculum. Med Teach.

[44] Spencer J (2003). ABC of learning and teaching in medicine: learning and teaching in the clinical environment. Br Med J.

[45] Rolfe IE, Sanson‐Fisher RW (2002). Translating learning principles into practice: a new strategy for learning clinical skills. Med Educ.

[46] Carlson E, Wann-Hansson C, Pilhammar E (2009). Teaching during clinical practice: strategies and techniques used by preceptors in nursing education. Nurse Educ Today.

[47] Berg KO, Wood-Dauphinee SL, Williams JI, Maki B (1992). Measuring balance in the elderly: validation of an instrument. Can J Public Health.

[48] Leech NL, Onwuegbuzie AJ (2011). Beyond constant comparison qualitative data analysis: using NVivo. Sch Psychol Q.

[49] Hsieh H-F, Shannon SE (2005). Three approaches to qualitative content analysis. Qual Health Res.

[50] Neuman WL (2006). Social research methods: qualitative and quantitative approaches.

[51] MacQueen KM, McLellan E, Kay K, Milstein B (1998). Codebook development for team-based qualitative analysis. Cultural Anthropology Methods.

[52] Guest G, Bunce A, Johnson L (2006). How many interviews are enough? An experiment with data saturation and variability. Field Methods.

[53] Kelly MA, Hager P, Gallagher R (2014). What matters most? Students’ rankings of simulation components that contribute to clinical judgment. J Nurs Educ.

[54] Sallis RE (2009). Exercise is medicine and physicians need to prescribe it!. Br J Sports Med.

[55] Abdulla A, Adams N, Bone M, Elliott AM, Gaffin J, Jones D (2013). Guidance on the management of pain in older people. Age Ageing.

[56] Campbell AJ (2009). Assertive screening: Health checks prior to exercise programmes in older people. Br J Sports Med.

[57] Franco MR, Pereira LSM, Ferreira PH (2014). Exercise interventions for preventing falls in older people living in the community. Br J Sports Med.

